# Adenosine deaminase as a marker for the severity of infectious mononucleosis secondary to EBV in children

**DOI:** 10.1186/s12879-022-07150-7

**Published:** 2022-02-21

**Authors:** Ting Shi, Jungen Li, Yuzhu Miao, Linlin Huang, Jianmei Tian

**Affiliations:** 1 Children’s Hospital of Soochow University, 303 Jingde Road, Suzhou, 215000 Jiangsu China; 2 The First Affiliated Hospital of Soochow University, 188 Shizi Road, Suzhou, Jiangsu 215000 China; 3 Pediatric intensive care unit, Children’s Hospital of Soochow University, 303 Jingde Road, Suzhou, 215000 Jiangsu China; 4 Department of Infectious Diseases, Children’s Hospital of Soochow University, 303 Jingde Road, Suzhou, 215000 Jiangsu China

**Keywords:** Infectious mononucleosis, Adenosine deaminase, Alanine aminotransferase

## Abstract

**Background:**

Infectious mononucleosis, a common disease in children and young adults, is often accompanied by elevated transaminase levels and rarely, liver failure. This study aimed to determine whether adenosine deaminase is a marker of severity in children with infectious mononucleosis, especially those with elevated alanine transaminase levels.

**Methods:**

This case-control study was conducted at the Children’s Hospital of Soochow University. A total of 104 children with infectious mononucleosis and 50 controls with other acute infections and fever, tonsillitis, or lymphadenitis, were enrolled in the study. Among the 104 children with infectious mononucleosis, 54 had normal alanine transaminase levels and 50 had elevated alanine transaminase levels. The children’s clinical and laboratory data were analyzed to assess the diagnostic value of adenosine deaminase in the three groups.

**Results:**

The adenosine deaminase level in the infectious mononucleosis group was significantly higher than that in the control group (*P* < 0.001). The adenosine deaminase levels were highly correlated with lymphocyte count, CD3^+^CD8^+^ T cells (%), CD4^+^/CD8^+^ ratio, and CD3^−^CD19^+^ (%) (r > 0.7, *P* < 0.01). The sensitivity and specificity of adenosine deaminase in predicting children with infectious mononucleosis were 97.1% and 94.0%, respectively. Furthermore, multivariate regression analysis revealed that adenosine deaminase level was a risk factor for elevated alanine transaminase in children with infectious mononucleosis.

**Conclusions:**

Adenosine deaminase may be a marker of the severity of infectious mononucleosis in children, and a predictor of elevated alanine transaminase in children with infectious mononucleosis.

## Background

Infectious mononucleosis (IM) is caused by acute Epstein-Barr virus (EBV) infection and presents with a classical triad of fever, pharyngitis, and lymphadenopathy [1]. It occurs mostly in children, adolescents, and young adults. In European and American countries, it mainly affects adolescents and adults aged 10–30 years [2]; however, in China, it usually occurs in children aged 4–6 years [3]. An elevated transaminase level is one of the most common features of IM. Several studies [4–6] have shown that transaminase levels are elevated in up to 80–90% of cases, while jaundice is seen in about 5% of cases. There are also rare cases where severe hepatitis or acute hepatic failure develop [6, 7].

Adenosine deaminase (ADA) is a purine metabolism enzyme encoded by the ADA gene. ADA mutations can lead to severe combined immune deficiency [8]. A large number of studies have confirmed that ADA plays an important role in the growth and differentiation of lymphocytes and macrophages, and is considered a marker of T lymphocyte-mediated cellular immunity [9]. Elevated ADA levels can be observed in autoimmune diseases, acquired immune deficiency syndrome, and tumors, and its level is closely related to the severity of these diseases [10, 11]. Previous research has shown that ADA levels can predict disease severity in individuals with hepatitis B, hepatitis C, and autoimmune hepatitis [12]. Although ADA is known to be associated with numerous diseases, its utility in assessing disease severity in pediatric patients with IM has not been determined.

The aim of this study was to explore the pathophysiology of ADA in children with IM and concurrently elevated ALT, to assess whether ADA measurement is useful for assisting clinicians in the timely diagnosis of IM and judgment of disease severity.

## Materials and methods

### Patient characteristics

This case-control study was conducted at the Children’s Hospital of Soochow University. Children with a disease onset > 5 days previously, mixed infections, autoimmune diseases and chronic diseases were excluded. The criteria of IM were as follows [12]: (1) presence of at least three of the following clinical manifestations: fever, pharyngitis, cervical lymphadenopathy, splenomegaly, eyelid edema and hepatomegaly; (2) IgM to EBV viral capsid antigen (VCA-IgM) and IgG to EBV capsid antigen (VCA-IgG) positive, with absence of the antibody to EB nuclear antigen (EBNA); and (3) exclusion of other viral infections such as human immunodeficiency virus, cytomegalovirus, hepatotropic virus and herpes simplex virus. The inclusion criteria of the control group were negative EBV-specific antibody and plasma EBV-DNA polymerase chain reaction (PCR) test results, disease onset < 5 days previously, and no history of chronic infectious diseases, immune system diseases and use of immunomodulators in the past 14 days. On admission to the hospital, the blood samples were collected for testing.

### Routine complete blood count, alanine transaminase, adenosine deaminase and immunoglobulin G, M, and A assays

Routine blood count of venous blood from participants was performed on the type BC-5310 instrument (Shenzhen Mindray Biomedical Electronics Co., Ltd). Serum ALT and ADA levels were measured using a lactate dehydrogenase assay (Beijing Strong Biotechnologies, Inc.) and peroxidase assay (test kit from Meikang Biotechnology Co., Ltd), respectively. Both were detected using a HITACHI 7180 biomedical analyzer. Immunoglobulin G (IgG), M (IgM), and A (IgA) were detected using a turbidimetric inhibition immunoassay. Anti-human IgG/IgM/IgA antibody (Orion Diagnostica Oy) was added to a certain proportion of the sample and buffer, which produced an agglutination reaction with IgG/IgM/IgA in the sample, resulting in an increase in the turbidity of the mixture. A Konelab clinical chemistry analyzer was used to detect turbidity at 340 nm wavelength.

### Flow cytometry

Lymphocyte subsets, including T cells (CD3^+^), helper T cells (CD3^+^CD4^+^), killer T cells (CD3^+^CD8^+^), natural killer cells (CD3^−^CD (16^+^56)^+^), B cells (CD3^−^CD19^+^), and activated B cells (CD19^+^CD23^+^) were detected using flow cytometry. Peripheral whole blood samples were labeled with antibodies including anti-CD3-fluorescein isothiocyanate, anti-CD16^+^56^−^ phycoerythrin, anti-CD45-peridin chlorophyll alpha protein-cyanin5.5, anti-CD4-phycoerythrin cyanin 7, anti-CD19-APC, anti-CD23-Fc EpsilonR II, and anti-CD8-allophycocyanin-cyanin7. The samples were centrifuged and kept in the dark at room temperature for 15 min. Each sample was analyzed using a multi-color flow cytometer (BD FACSCanto II) according to the manufacturer’s instructions.

### Indirect immunofluorescence assay (IIF) for EBV-specific antibodies

The anti-EBV-VCA IgG /IgM, anti-EBV-early antigen (EA) IgG and anti-EBNA IgG IIF kits (EUROIMMUN, Lübeck, Germany) was used for testing the EBV-VCA, EA and EBNA in serum. All steps were carried out according to the manufacturer’s instructions. The antibody affinity was determined by comparing the serum fluorescence intensities of children treated with and without urea treatment. The difference in fluorescence intensity was ≥ 2 for low affinity, and < 2 for high affinity.

### Plasma Epstein-Barr virus-DNA polymerase chain reaction assay

Plasma EB viral load was determined by polymerase chain reaction, and EBV nucleic acid quantitative detection kit came from Shengxiang Biotechnology Co., Ltd, Hunan, China. Amplification and detection were performed on the LightCycler 480II instrument (Roche, Basel, Switzerland) following the manufacturer’s instructions. Copy numbers were calculated by comparing the cycle threshold (Ct) of the specimens to the standard curve. EBV positivity was defined as a Ct value ≤ 39 (DNA copy number > 400 copies/mL).

All peripheral blood samples were collected within 24 h of admission and sent to the laboratory for analysis immediately.

### Statistical analysis

The Shapiro-Wilk normality test was used to determine whether continuous variables were normally distributed. Values were expressed as the mean ± standard deviation or median and interquartile range. The Mann-Whitney U-test and Kruskal-Wallis test were used for non-normally distributed data. Student’s t-test and analysis of variance (ANOVA) were used for normally distributed variables. Categorical variables were reported as frequency (%) and the frequency in different groups was compared using Chi-squared test or Fisher’s exact test. Spearman correlation analysis was used to determine the correlation between discrete variables. Logistic regression analysis was used to determine odds ratios (ORs) with 95% confidence intervals (CIs). Receiver-operating characteristic (ROC) curve analysis was used to assess the diagnostic accuracy of adenosine deaminase in children with IM. The cut-off value for ADA was determined using Youden’s index. All statistical analyses were performed using SPSS version 25.0 (IBM Corp., Armonk, NY, USA). *P*-values < 0.05 were considered to be statistically significant.

## Results

### Clinical characteristics of the infectious mononucleosis group and the control group

A total of 104 patients with IM and 50 control children with acute infectious diseases were enrolled in the study. There were 47 males and 57 females, with median age of 2.9 (1.9–4.1) years in the IM group. Among them, 54 children had normal ALT levels (IM1 group) and 50 had elevated ALT levels (IM2 group). There were 50 patients in the control group, 27 males and 23 females, with a median age of 3.2 years. There were no statistically significant differences in sex, age, and course of disease between the IM group and the control group. The lymphocyte count, ADA, IgA, IgG, IgM, CD3^+^ (%), and CD3^+^CD8^+^ (%) in children with IM were all significantly higher in the children with IM than in the controls (*P* < 0.001) (Table 1). However, the values of CD3^+^CD4^+^ (%), CD4^+^/CD8^+^ ratio, CD3^−^CD19+ (%), and CD19^+^CD23^+^ (%) were lower in the IM group (*P* < 0.001), and CD19 + CD56+ (%) did not differ significantly between the two groups (*P* = 0.616).

**Table 1:** Comparison of Laboratory Findings between IM group and non-IM group

Parameters	IM group (n = 104)	Non-IM group (n = 50)	*P*
Sex (male)	47 (45.2)	27 (54.0)	0.306
Age (years)	2.9 (1.9–4.1)	3.2 (2.2–5.1)	0.207
Disease course (days)	3.0 (3.0–4.0)	3.0 (2.8-4.0)	0.263
WBC (×10^9^/L)	16.4 (12.3–21.0)	13.7 (9.3–19.3)	0.049
Lymphocyte count(×10^9^/L)	10.6 (7.0–14.0)	3.4 (2.3–5.2)	< 0.001
ADA (U/L)	51.6 (44.5–66.5)	20.1 (17.1–23.8)	< 0.001
IgA (g/L)	1.4 (1.0–1.9)	0.8 (0.5–1.2)	< 0.001
IgG (g/L)	11.3 ± 2.8	7.7 ± 2.2	< 0.001
IgM (g/L)	1.6 (1.3–2.2)	1.1 (0.8–1.6)	< 0.001
CD3+ (%)	79.1 (72.6–82.8)	63.8 (54.1–71.8)	< 0.001
CD3 + CD4+ (%)	17.6 (13.3–21.7)	32.1 (24.8–39.2)	< 0.001
CD3 + CD8+ (%)	53.9 ± 11.0	25.0 ± 8.0	< 0.001
CD4+/CD8+	0.3 (0.2-0.475)	1.2 (1.0-1.7)	< 0.001
CD3-CD (16 + 56) + (%)	11.9 (8.1–16.9)	11.1 (6.7–16.1)	0.616
CD3-CD19+ (%)	7.8 (5.2–9.8)	20.9 (16.0–29.8)	< 0.001
CD19 + CD23+ (%)	4.0 (2.4–6.3)	9.9 (6.8–12.7)	< 0.001

The data presented as median [interquartile range], mean ± standard deviation and n (%). The univariate analyses were performed using Mann-Whitney U-test for skewed distribution variables, t-test for normal distribution variables and the chi-square test for categorical variables. WBC, white blood cell; ADA, adenosine deaminase; IgA, immunoglobulin A; IgG, immunoglobulin G; IgM, immunoglobulin M

### Correlation of adenosine deaminase with laboratory parameters

**Fig. 1:**
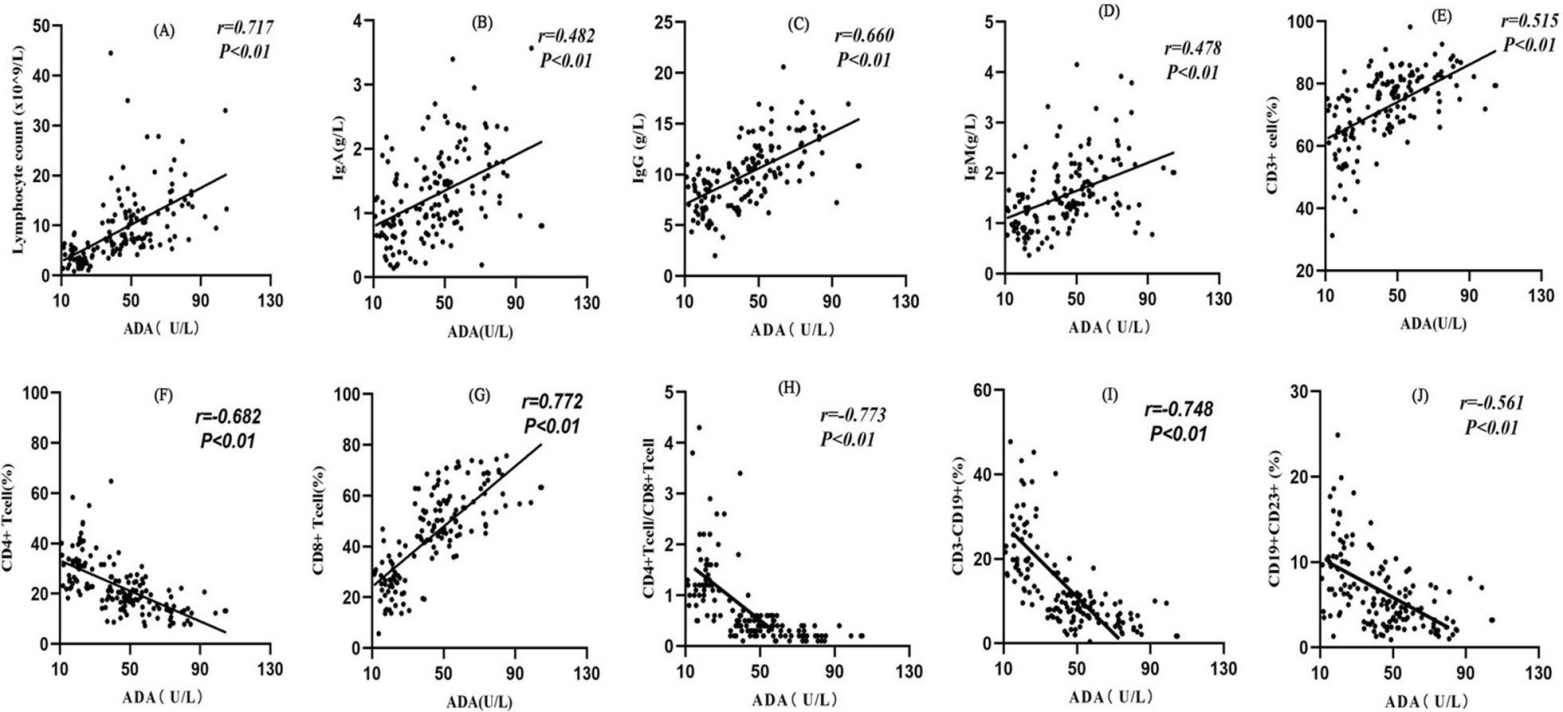
Correlation between adenosine deaminase and immune cells/globulin

The ADA levels were highly correlated with the lymphocyte count, CD3^+^CD8^+^T cells (%), CD4^+^/CD8^+^ ratio, and CD3^−^CD19^+^ (%) (r > 0.7, *p* < 0.01) (Fig. 1). The lymphocyte count and CD3^+^CD8^+^ T cells (%) were positively correlated with the ADA level, while the CD4^+^/CD8^+^ ratio and CD3^−^CD19^+^ (%) were negatively correlated with the ADA level.

### Diagnostic accuracy of adenosine deaminase in infectious mononucleosis

**Fig. 2:**
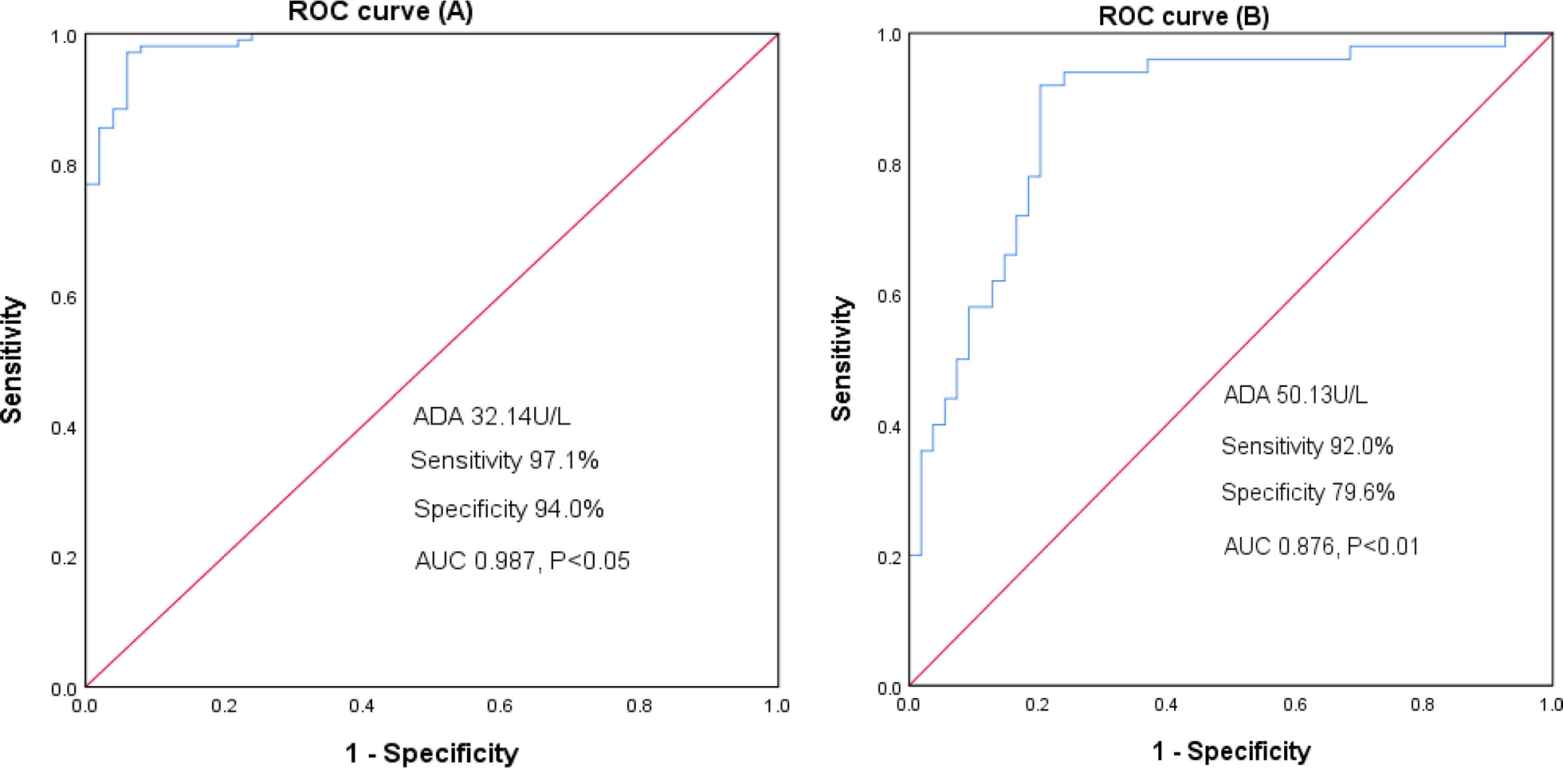
The diagnostic value of adenosine deaminase in infectious mononucleosis. **A** The diagnostic value of adenosine deaminase for IM. **B** the diagnostic value of adenosine deaminase for IM with elevated alanine aminotransferase

The ADA level had a high diagnostic accuracy for IM (Fig. 2). The cut-off value for ADA was set at 32.14 U/L, the sensitivity was 97.1%, the specificity was 94.0%, and the area under the curve was 0.987 (*P* < 0.05).

### Predictive value of adenosine deaminase for alanine transaminase in patients with infectious mononucleosis

**Fig. 3:**
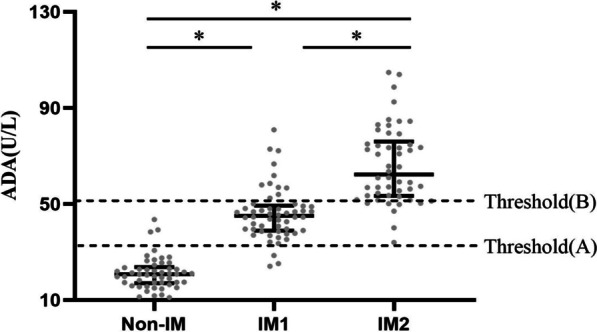
The level (Interquartile range) of adenosine deaminase in the non-IM group, IM1 group (IM with normal ALT) and IM2 group (IM with elevated ALT). Threshold (A) for diagnosing IM and threshold (B) for diagnosing IM with elevated ALT. IM, Infectious mononucleosis; ALT Alanine aminotransferase; *p < 0.05

There were no statistically significant differences in the clinical manifestations between IM patients with elevated (IM2 group) and normal ALT (IM1 group), but the incidence of cervical lymphadenopathy, hepatomegaly, splenomegaly, and puffy eyelids in the IM1 and IM2 groups were significantly higher than that in the non-IM group (Table 2). Plasma DNA load, ADA, IgG, and CD3 + CD8+(%) were differed significantly between the IM1group and the IM2 group (Table 3). Univariate and multivariable logistic regression analyses found that ADA level was an independent risk factor for elevated ALT (adjusted OR: 1.124, 95% CI 1.063–1.189) (Table 4). ROC curve analysis was used to evaluate the diagnostic value of ADA for predicting ALT (Fig. 2). The sensitivity and specificity were 92.0% and 79.6%, respectively. ADA had a high diagnostic accuracy in predicting IM (cut-off value, 32.14 U/L) and predicting elevated ALT (cut-off value: 50.13 U/L) (Fig. 3).

**Table 2:** Clinical Characteristics of IM patients with normal and elevated ALT

Parameters	IM1 group (n = 54)	IM2 group (n = 50)	Controls (n = 50)	*P*
Sex (male)	23 (42.6)	24 (48.0)	27 (54.0)	0.508
Age (years)	3.2 (1.9–4.4)	2.8 (2.0–4.0)	3.2 (2.2–5.1)	0.377
Disease course (days)	3.0 (3.0–4.0)	4.0 (3.0–4.0)	3.0 (2.8–4.0)	0.148
Peak of fever (℃)	39.2 (38.7–39.5)	39.1 (38.9–39.5)	39.3 (39.0–40.0)	0.07
Pharyngitis	47 (87.0)	41 (82.0)	42 (84.0)	0.775
Cervical lymphadenopathy	54 (100.0)^a^	49 (98.0)^a^	30(60%)^b^	< 0.01
Hepatomegaly	15 (27.8)^a^	16 (32.0)^a^	(0.0%) ^b^	< 0.01
Splenomegaly	18 (33.3)^a^	21 (42.0)^a^	(0.0%)^b^	< 0.01
Rash	9 (16.7)	7 (14.0)	5 (10%)	0.610
Puffy eyelid	39 (72.2) ^a^	34 (68.0)^a^	(0.0%)^b^	< 0.01

The data are reported as median (interquartile range) or n (%). The univariate analyses were performed using Kruskal-Wallis test for skewed distributed data and the chi-square test or fisher′s exact test for categorical variables. IM1 group represents the normal ALT group, IM2 group represents elevated ALT group. *P* < 0.05 between a and b

**Table 3:** Laboratory Findings of IM patients with normal and elevated ALT

Parameters	IM1 group (n = 54)	IM2 group (n = 50)	Controls (n = 50)	*P*
WBC (×10^9^/L)	15.3 (11.4–19.5)^ab^	17.2 (12.8–23.3)^a^	13.7 (9.3–19.3)^b^	0.037
Lymphocyte count(×10^9^/L)	10.4 (6.4–12.7)^a^	11.8 (7.4–16.1)^a^	3.4 (2.3–5.2)^b^	< 0.01
Plasma EBV-DNA lg(copies/ml)	3.7 ± 0.6a	4.1 ± 0.7b	–	0.003
ADA (U/L)	45.1 (38.9–49.4)^a^	62.3 (53.4–79.3)^b^	20.1 (17.1–23.8)^c^	< 0.01
IgA (g/L)	1.3 (1.0–1.8) ^a^	1.5 (1.0–2.0)^a^	0.8 (0.5–1.2)^b^	< 0.01
IgG (g/L)	10.4 ± 2.5^a^	12.2 ± 2.8^b^	7.7 ± 2.2^c^	< 0.05
IgM (g/L)	1.4 (1.1–2.0)^a^	1.8 (1.4–2.2)^a^	1.1 (0.8–1.6)^b^	< 0.01
CD3+ (%)	78.1 (71.0–82.8)^a^	79.4 (75.0–82.7)^a^	63.8 (54.1–71.8)^b^	< 0.01
CD3+ CD4+ (%)	19.1 (14.5–23.3)^a^	15.6 (12.6–20.6)^a^	32.1 (24.8–39.2)^b^	< 0,01
CD3+ CD8+ (%)	51.6 ± 10.1^a^	56.3 ± 11.4^b^	25.0 ± 8.0^c^	< 0.05
CD4+/CD8+	0.4 (0.3–0.5)^a^	0.3 (0.2–0.4)^a^	1.2 (1.0–1.7)^b^	< 0.01
CD3-CD(16 + 56)+(%)	11.8 (7.7–16.9)	11.9 (9.0–17.0)	11.1 (6.7–16.1)	0.830
CD3-CD19+ (%)	8.1 (5.4–11.8)^a^	7.1 (4.4–9.4)^a^	20.9(16.0–29.8)^b^	< 0.01
CD19 + CD23+ (%)	4.4 (2.4–6.4)^a^	3.5 (2.4–6.1)^a^	9.9 (6.8–12.7)^b^	< 0.01

The data presented as median [interquartile range], mean ± standard deviation and n (%). The univariate analyses were performed using Kruskal-Wallis test for skewed distribution variables, and ANOVA for normal distribution variables. IM1 group represents the normal ALT group, IM2 group represents elevated ALT group. WBC, white blood cell; ADA, denosine deaminase; IgA, immunoglobulin A; IgG, immunoglobulin G; IgM, immunoglobulin M. *P*< 0.05 between a, b and c

**Table 4:** Factors associated with elevated ALT in IM patients (multivariate analysis)

Variable	Model 1		Model 2		Model 3	
	OR (95%CI)	*P*	OR (95%CI)	*P*	OR (95%CI)	*P*
Plasma EBV-DNA lg(copies/ml)	2.551 (1.325–4.912)	0.005	2.650 (1.358–5.173)	0.004	1.370 (0.698–2.687)	0.361
ADA(U/L)	1.125 (1.073–1.179)	< 0.01	1.129 (1.075–1.185)	< 0.001	1.124 (1.063–1.189)	< 0.001
IgG(g/L)	1.297 (1.103–1.525)	0.002	1.349 (1.130–1.610)	0.001	1.099 (0.879–1.375)	0.406
CD8+ (%)	1.042 (1.004–1.082)	0.030	1.050 (1.008–1.093)	0.019	0.970 (0.917–1.026)	0.294

ALT, alanine aminotransferase; IM, infectious Mononucleosis; ADA, adenosine deaminase; IgG, immunoglobulin G; OR, odds ratio; CI, confidence interval. Model 1 was not adjusted. Model 2 was adjusted for age and sex. Model 3 was adjusted as Model 2 + Plasma EBV-DNA, ADA, IgG, CD8+ (%)

## Discussion

EBV is prevalent worldwide. The symptoms of EBV infection in children are often atypical [13], and the diagnosis relies mainly on serological assays [14]. However, due to the immature immune system of children and the delay in developing IgM antibodies, it is easy clinically misdiagnose IM in children and to miss the diagnosis. In this study, ADA was significantly higher in children with IM than in those with acute infectious diseases caused by other pathogens, and it has a high diagnostic accuracy for identifying children with IM. ADA also has a high predictive value for predicting which children had high ALT in the early stages of IM, which is often asymptomatic. Therefore, ADA can not only help clinicians diagnose IM early, but also indirectly reflect the severity of the disease.

IM is caused by acute EBV infection and presents with a classical triad of fever, pharyngitis, and lymphadenopathy [1], which are difficult to distinguish from febrile diseases caused by other pathogens. Previous studies [15, 16] have confirmed that the absolute lymphocyte count is significantly elevated in children with IM, especially CD8 + T cells, and that the CD4/CD8 ratio becomes inverted. This is consistent with the results of this study. In addition, this study found that the incidence of cervical lymphadenopathy, hepatomegaly, splenomegaly, and puffy eyelids, and the levels of immunoglobulins (IgA, IgG, and IgM) in children with IM was higher than that in children with infectious diseases caused by other pathogens.

Previous studies have shown that ADA plays an important role in the growth and differentiation of lymphocytes and macrophages [9], and it was elevated in children with IM, but its mechanism has not been described [17]. This study also found that ADA levels were significantly increased in children with IM, and that there was a high correlation between the ADA level and the absolute lymphocyte count.

ROC curve analysis showed that ADA had a high diagnostic accuracy for distinguishing children with IM from children with acute febrile diseases caused by other pathogens. The most common feature of infectious mononucleosis was elevated transaminases [4–6]. In this study, there was no difference in clinical manifestations of IM between children with elevated and normal ALT levels, but the viral load, ADA, CD8^+^ T cell (%), and IgG level were higher in children with elevated ALT levels. Previous studies have found that the absolute lymphocyte count is an indicator of Epstein-Barr virus-related liver damage [18], and that the EBV-DNA copy number is directly proportional to the severity of IM [19].

The mechanism of EBV hepatitis is thought to be that EBV infected and activated CD8 + T-cells accumulate in the liver and the products of the EBV-infected CD8 + T cells or infiltrating cytotoxic T lymphocytes were interferon-γ, tumor necrosis factor and Fas ligand, which can destroy hepatocytes [20–23]. In this study, the ADA level was closely related to the ALT level and had a good predictive value for ALT in the children with IM. In addition, it had a high diagnostic value for IM even when EBV-related antibodies were not produced in the early stage. Therefore, ADA could play a useful role in the diagnosis of IM and the prediction of the severity of the disease in the early stages of the disease.

Our study has several limitations. First, liver biopsy, to better characterize the liver pathology in patients with IM was lacking. Second, we only studied ADA and ALT at a single point in time early in the course of disease.

## Conclusions

ADA had a high diagnostic accuracy for identifying IM in children with acute febrile diseases. ADA was highly correlated with the lymphocyte count in children with IM, especially CD8 + T cells. In addition, the ADA level indirectly reflected the severity of IM in children in the early stages, especially those with elevated ALT levels. These results reveal that measuring ADA in children with suspected IM can play a useful role in helping clinicians diagnose IM early and in predicting the severity of the disease.

## Data Availability

The data used in this study are available from the corresponding author on reasonable request.

## Abbreviations

IM: Infectious mononucleosis
ALT: Alanine aminotransferase
OR: Odds ratio
CI: Confidence interval
ROC: Receiver operating characteristic
WBC: White blood cell
ADA: Adenosine deaminase
IgA: Immunoglobulin A
IgG: Immunoglobulin G
IgM: Immunoglobulin M
ADA: Adenosine deaminase
